# Asymmetric post-translational modifications regulate the nuclear translocation of STAT3 homodimers in response to leukemia inhibitory factor

**DOI:** 10.1007/s13402-023-00911-9

**Published:** 2023-12-27

**Authors:** Mickael Diallo, Constança Pimenta, Fernanda Murtinheira, Daniela Martins-Alves, Francisco R. Pinto, André Abrantes da Costa, Ricardo Letra-Vilela, Vanesa Martin, Carmen Rodriguez, Mário S. Rodrigues, Federico Herrera

**Affiliations:** 1 BioISI – Instituto de Biosistemas e Ciências Integrativas, Faculdade de Ciências da Universidade de Lisboa, Lisbon, 1749-016 Portugal; 2 MOSTMICRO Research Unit, Instituto de Tecnologia Química e Biológica (ITQB-NOVA), Universidade Nova de Lisboa, Oeiras, Portugal; 3 Departamento de Morfología y Biología Celular, Facultad de Medicina, University of Oviedo, c/Julian Claveria, Oviedo, 33006 Spain; 4 Instituto Universitario de Oncología del Principado de Asturias (IUOPA), Oviedo, Spain

**Keywords:** STAT3, Homodimerization, Post-translational modifications, Leukemia inhibitory factor

## Abstract

STAT3 is a pleiotropic transcription factor overactivated in 70% of solid tumours. We have recently reported that inactivating mutations on residues susceptible to post-translational modifications (PTMs) in only one of the monomers (i.e. asymmetric) caused changes in the cellular distribution of STAT3 homodimers. Here, we used more controlled experimental conditions, i.e. without the interference of endogenous STAT3 (STAT3-/- HeLa cells) and in the presence of a defined cytokine stimulus (Leukemia Inhibitory Factor, LIF), to provide further evidence that asymmetric PTMs affect the nuclear translocation of STAT3 homodimers. Time-lapse microscopy for 20 min after LIF stimulation showed that S727 dephosphorylation (S727A) and K685 inactivation (K685R) slightly enhanced the nuclear translocation of STAT3 homodimers, while K49 inactivation (K49R) delayed STAT3 nuclear translocation. Our findings suggest that asymmetrically modified STAT3 homodimers could be a new level of STAT3 regulation and, therefore, a potential target for cancer therapy.

**Supplementary Information:**

The online version contains supplementary material available at 10.1007/s13402-023-00911-9.

Signal Transducer and Activator of Transcription 3 (STAT3) is a pleiotropic transcription factor playing essential roles in normal development, immunity, response to stress/damage and cancer [1, 2]. Latent STAT3 monomers and homodimers shuttle between the cytoplasm and the nucleus [3]. Activation of STAT3 by various types of extracellular signals, such as the IL-6 family of cytokines, triggers the accumulation of STAT3 dimers in the nucleus and the transcription of a specific set of genes [4, 5]. Phosphorylation at Y705 is commonly considered the canonical, rate-limiting step for STAT3 dimerization, nuclear translocation, DNA binding and transcriptional activation [6, 7]. However, unphosphorylated STAT3 (at Y705) also dimerizes, is present in the nucleus, binds to DNA and activates a specific set of genes different from Y705-phosphorylated STAT3 [8–11]. Phosphorylation at S727, or acetylation, methylation, ubiquitination or sumoylation at K49 or K685 are among the best studied post-translational modifications (PTMs) in STAT3, and they modulate both canonical and non-canonical functions of this transcription factor [12]. However, more than 80 PTMs are described for STAT3 [2], and their function and stoichiometry in the presence or absence of stimulating cytokines remain poorly understood.

Considering the high number of possible PTMs, it is unlikely that STAT3 homodimers are identical in terms of PTM patterns. We have recently shown that asymmetric post-translational modifications can produce striking changes in the subcellular distribution of STAT3 homodimers [13]. However, these experiments were carried out in HeLa cells expressing endogenous STAT3; and in the presence of Fetal Bovine Serum (FBS), containing unidentified signalling molecules with effects on the STAT3 pathway.

Here, we corrected these issues to confirm or reject the original hypothesis. We developed a STAT3-/- HeLa strain using commercially available CRISPR/Cas9 (sc-400,027, Santa Cruz Biotechnologies, Dallas, TX), cell sorting and clonal selection procedures described elsewhere [14]. Cells were maintained and seeded as described previously [13, 14]. This new HeLa strain does not express endogenous STAT3 (Fig. 1A). Transfection with one of our VN-STAT3α BiFC construct achieves expression levels similar to the original HeLa cells (ATCC, Ref. CRM-CLL-2)(Fig. 1A). For the following experiments, cells were transfected with two complementary constructs of our Venus-STAT3α bimolecular fluorescence complementation (BiFC) system, a pair of constructs containing complementary fragments of the Venus fluorescence reporter (VN and VC, Fig. 1B), in a 1:1 proportion. This system allows the visualization of STAT3 homodimers in living cells [13]. PTM-resistant constructs were produced by PCR-based site-directed mutagenesis, as previously described [13], and the constructs were deposited in Addgene (https://www.addgene.org/). Sixteen hours after transfection, FBS-containing medium was substituted by serum-free medium 2 h before addition of Leukemia Inhibitory Factor (LIF, 200 ng/ml), and we recorded time-lapse series of randomly selected cells for 20 min after cytokine addition by widefield fluorescence microscopy. Images were later analyzed by means of ImageJ/FIJI Software (See Supplementary Methods for further details).

STAT3-/- HeLa cells transfected with a symmetric combination of two wild-type (WT) monomers showed a relatively homogenous nucleocytoplasmic distribution. As expected, we observed a rapid, cytokine-dependent translocation of wild-type STAT3 homodimers from the cytosol to the nucleus and probably the membrane (punctae)(Fig. 1C). Nuclear accumulation is mostly dependent on STAT3 phosphorylation at Y705 since the symmetric Y705F STAT3 homodimers barely changes their intracellular distribution. However, asymmetric WT + Y705F STAT3 homodimers were also able to accumulate in the nucleus in response to LIF, suggesting that phosphorylation in only one of the STAT3 monomers is sufficient for nuclear translocation of the dimers (Fig. 1D). Immunoblotting assays indicated that this is also true for asymmetric PTMs at K49, K685 and S727 (Suppl. Figure 1). Nuclear translocation of WT + WT and WT + Y705F STAT3 dimers grows linearly before it reaches a plateau 10–13 min after addition of LIF, being the asymmetric dimers slightly but significantly delayed at 5 min (Fig. 1D). However, phosphoresistant Y705F + Y705F dimers never achieved more than ~ 110% nucleocytoplasmic ratio. These data suggest that phosphorylation at Y705 of one STAT3 monomer can be enough to ensure the nuclear translocation of STAT3 dimers in response to LIF.

Unphosphorylated STAT3 (here mimicked as Y705F) is relevant for a number of newly found roles in cancer, but the role of additional PTMs in its function is barely understood [2]. We inactivated S727 phosphorylation (S727A) on Y705F-STAT3 BiFC constructs to understand its possible contribution to the behavior of Y705-unphosphorylated STAT3 dimers. In resting state, an additional S727A mutation did not change the previously observed homogenous nucleocytoplasmic distribution of STAT3 dimers (Fig. 1D). LIF induced a rapid nuclear translocation of WT + Y705F/S727A STAT3 homodimers, similar to symmetric WT homodimers.

**Fig. 1:**
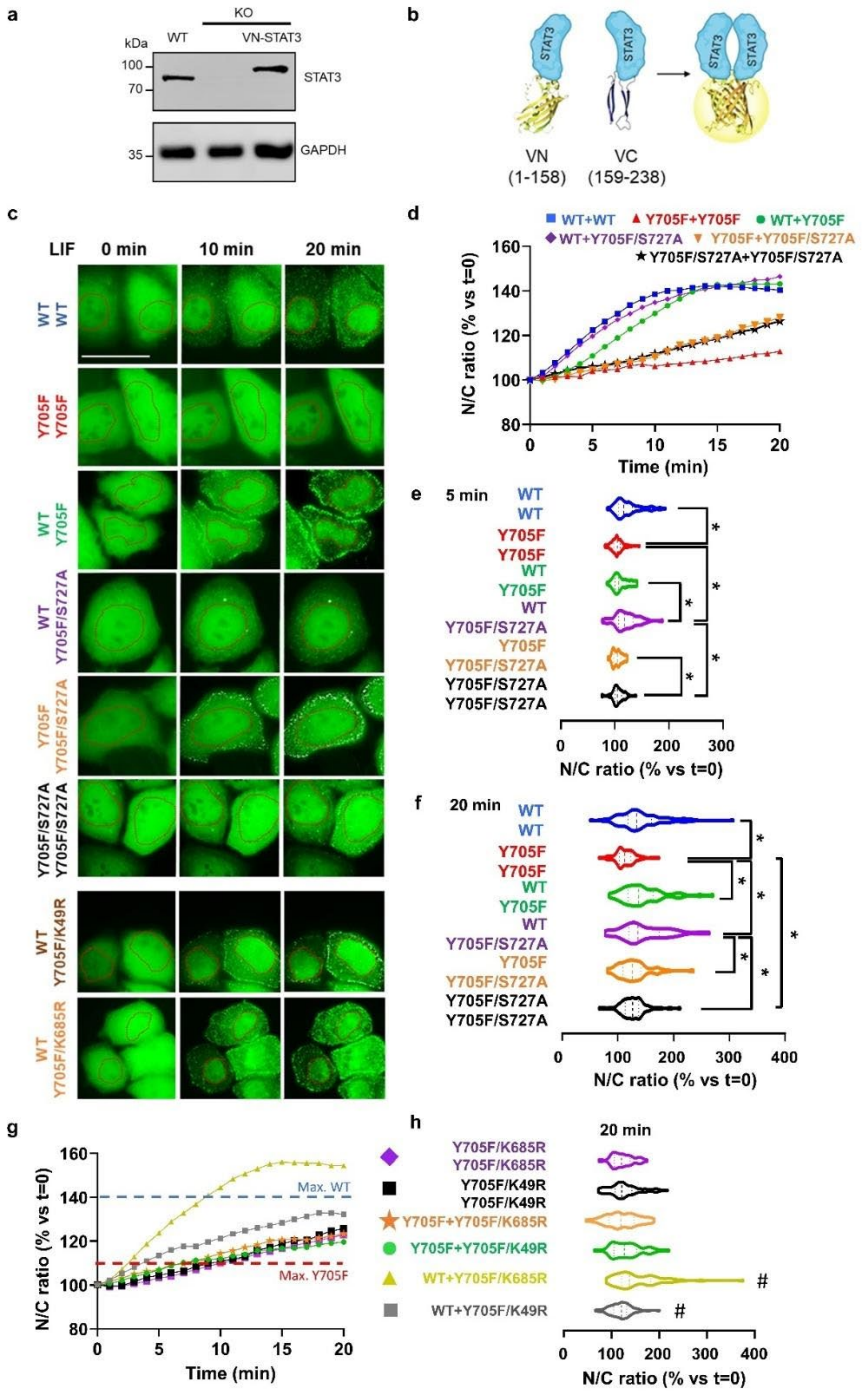
Asymmetric PTMs can modulate the subcellular localization of STAT3 homodimers upon cytokine stimulation. **a** Representative western blot images of total protein extracts from reference HeLa and STAT3-/- strains transfected with wild-type VN-STAT3 construct, expressing STAT3 levels similar to endogenous STAT3 in reference HeLa cells. **b** Our Venus-STAT3 BiFC system consists of two constructs where STAT3α was fused to either the N-terminus (VN, amino acids 1-158) or C-terminus (VC, amino acids 159–222) of the Venus fluorescent reporter. STAT3 dimerization brings the complementary Venus fragments together, restoring fluorescence. Fluorescence intensity is proportional to STAT3 homodimerization. **c** Representative frames from time-lapsed videos of STAT3-/- HeLa cells transfected with the indicated BiFC pairs of Venus-STAT3 constructs (green) at 0, 10 and 20 min of incubation with LIF (200 ng/ml). Red line marks the nuclear perimeter. Scale bar, 40 μm. **d** Average kinetic curves of LIF-induced nuclear translocation of STAT3 dimers in > 50 cells (Nr. of independent experiments: WT pair, N = 11; Y705F pair N = 5; any other pair N = 4). For each cell, the fluorescence intensity was measured in identically shaped and sized regions of interest (ROI) within nuclear and cytosol compartments. The kinetic evolution of the ratio between nuclear and cytosolic fluorescence intensity values was used as an indicator of STAT3 nuclear translocation. An identically shaped and sized ROI - within a region with no apparent fluorescence detected – was used to measure the background value. Background value was then subtracted from nuclear and cytosolic values before normalization versus t = 0. **e** and **f** Violin plots representing the sample distribution of N/C ratios at t = 5 and t = 20 for each combination. **g-h** Same as in **d-f** but with the indicated combinations carrying K49R or K685R mutations. Only t = 20 min was represented here, as it is the time with maximal differences between groups. Blue and red lines mark the maximum average response by the WT pair and the Y705F pair, respectively. Results were analyzed by means of One-way ANOVA, followed by a Tukey post-hoc test. n = number of analyzed cells: WT pair, 160; Y705F pair, 87; WT + Y705F, 56; WT + Y705F/K49R, 59; Y705F + Y705F/K49R, 100; Y705F/K49R + Y705F/K49R, 68; WT + Y705F/K685R, 64; Y705F + Y705F/K685R, 56; Y705F/K685R + Y705F/K685R, 60; WT + Y705F/S727A, 47; Y705F + Y705F/S727A, 52; Y705F/S727A + Y705F/S727A, 78. *, significant vs. each other, p < 0.05; #, significant vs. Y705F pair, p < 0.05

However, when phosphorylation was prevented in one or both residues in both monomers (Y705F + Y705F/S727A and Y705F/S727A + Y705F/S727A), nuclear translocation of STAT3 dimers was impaired. Still, there was some residual nuclear translocation higher than in the Y705F pair (Fig. 1D and F). These data suggest that S727 phosphorylation in only one STAT3 monomer is enough to retain in the cytoplasm those STAT3 dimers that were not phosphorylated at Y705 in response to cytokines.

Similar analyses were carried out in K49R and K685R mutants, which prevent any PTM in these lysines, further supporting our hypothesis that asymmetric PTMs could be relevant to the subcellular localization of STAT3 homodimers in response to cytokines. LIF induced significant nuclear translocation of WT + Y705F/K49R and WT + Y705F/K685R homodimers (Fig. 1G and H). However, a K49R in the non-phosphorylatable STAT3 monomer reduced nuclear translocation of STAT3 dimers (Fig. 1G). In turn, a K685R mutation increased nuclear accumulation compared to WT STAT3 homodimers and asymmetric WT + Y705F STAT3 homodimers (Fig. 1H). The symmetric combinations Y705F/K49R + Y705F/K49R and Y705F/K685R + Y705F/K685R and the asymmetric combinations Y705F + Y705F/K49R and Y705F + Y705F/K685R increased slightly nuclear accumulation versus symmetric Y705F + Y705F dimers, but the difference was not significant (Fig. 1G and H). K49 is in the N-terminus of the protein and K685 in the C-terminus, but both are relevant to STAT3 activity. K49 can be acetylated by p300/CBP [15] or methylated by EZH2 [12] and both events promote STAT3 activity. On the other hand, the role of K685 acetylation by p300/CPB is relatively controversial, as some groups reported enhancing/stabilizing effects [11, 16] while others described inhibition of STAT3-induced transcription [17]. Our results would support the former, and indicate that the role of PTMs on these two residues should be further studied in the context of non-canonical STAT3 functions mediated by unphosphorylated STAT3.

To understand whether asymmetric PTMs could have consequences in the expression profiles induced by LIF, total protein extracts from cells expressing three STAT3 dimer variants (WT-WT, WT-Y705F and Y705F-Y705F) with and without LIF stimulation for 24 h were analyzed by Mass Spectrometry (two technical replicates for each of the 6 experimental conditions). The lists of identified proteins are deposited in a public repository (10.5281/zenodo.8328034). Although these results should be considered with caution, they indicate that asymmetric phosphorylation at Y705 could produce a distinct expression profile (Fig. 2), only partially overlapping with phosphorylatable and non-phosphorylatable symmetric combinations. Such overlap suggests that part of the expression profiles associated to Y705-phosphorylated and unphosphorylated STAT3 could be mediated by partially phosphorylated STAT3 dimers.

Asymmetric post-translational modifications have been described for retroviral reverse transcriptases (RT). The active, mature HIV-1 RT results from post-translational cleavage of only one monomer in the p66 homodimer [18]. PTM asymmetry may confer a differential role between the two monomers in the same manner as heterodimers or ligand-bound homodimers [19]. For example, nuclear translocation of the STAT3-RelA heterodimer depends on the nuclear localization sequence (NLS) of STAT3, while DNA binding is described as RelA function [20]. Point mutations in one of the monomers in cAMP receptor protein (CRP) homodimers can change the allosteric ligation of cAMP to the dimers, but ligation of only one cAMP molecule to the dimer was enough to trigger DNA binding [19]. In the context of STAT3, there are several situations where STAT3 dimers could be formed by two slightly different STAT3 monomers with functional consequences, just as it happens with asymmetric PTMs. Several gain-of-function and dominant negative mutations in only one STAT3 allele were associated to human pathologies, suggesting that normal and mutant forms of STAT3 can coexist, dimerize and function in many instances [21, 22]. STAT3β is a non-pathogenic splicing isoform that coexist with the main STAT3α isoform (the one used in this study) with variable stoichiometry in different cells and tissues, also having consequences for human pathologies [23, 24]. Our current results confirm the hypothesis we recently launched [2, 13], indicating that the subcellular distribution of STAT3 homodimers is influenced by asymmetric PTMs in the absence of endogenous STAT3 and in response to cytokines. The stoichiometry of STAT3 PTMs, isoforms and mutations, and the contribution of asymmetric dimers to known STAT3 functions should be further studied.

**Fig. 2:**
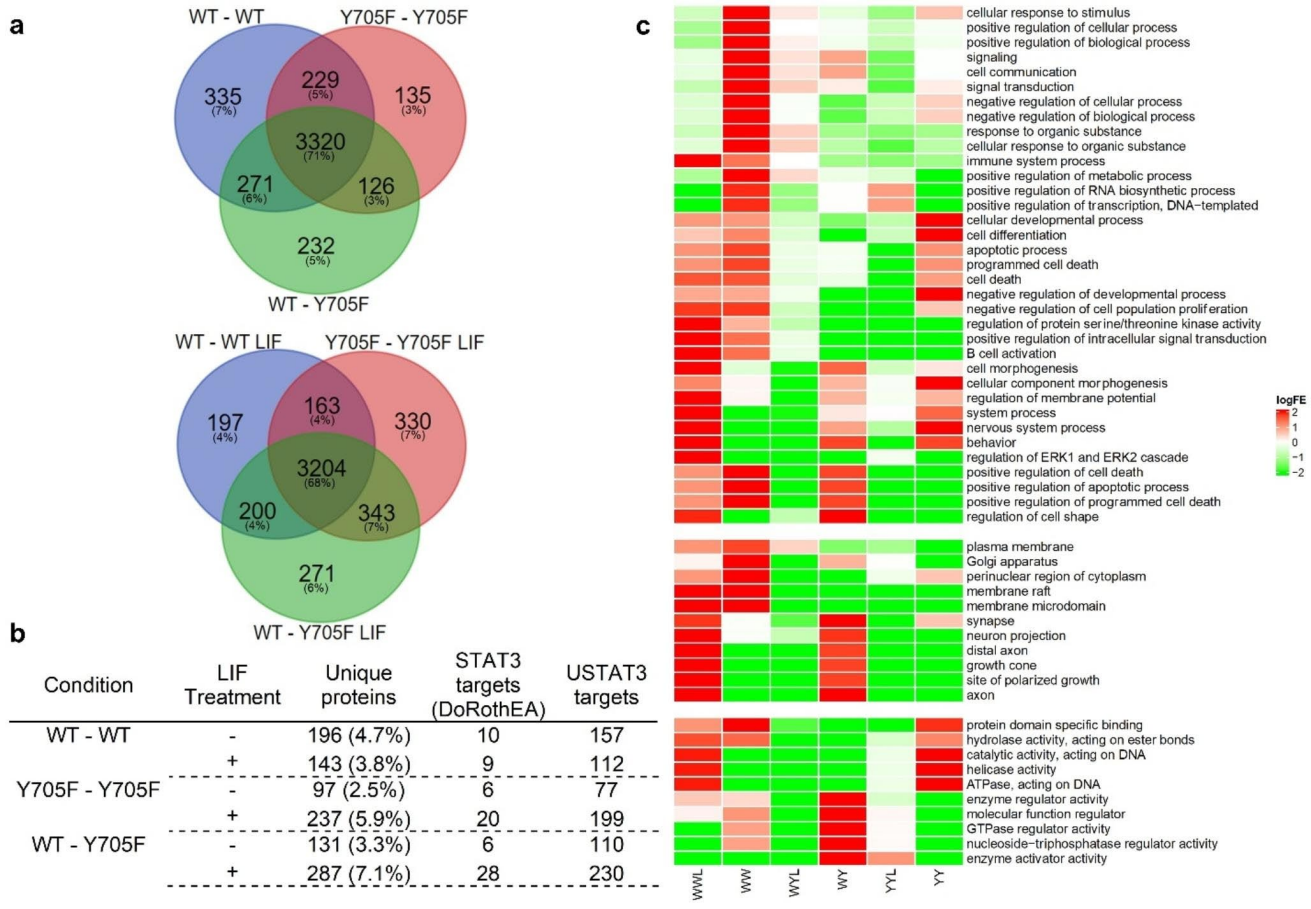
Expression of different STAT3 variants has impact on cell’s proteome and on the functional profile of regulated targets. **a** Venn diagrams describing the overlaps between the sets of identified proteins in cells expressing the three STAT3 variants (WT-WT, WT-Y705F and Y705F-Y705F) with and without LIF stimulation. Proteins identified uniquely in one condition are potentially specific targets of the corresponding STAT3 variant. **b** Table showing the number of proteins uniquely identified in each of the 6 conditions studied (3 STAT3 variants with and without LIF stimulation), and within those sets, how many are codified by genes known to be regulated by STAT3 according to the DoRothEA database or to unphosphorylated STAT3 (USTAT3) ChIP-seq data. **c** Heatmap representing GO terms significantly associated with targets of specific STAT3 variants with or without LIF stimulation. Color represents the logarithm (base 2) of the Fold Enrichment (logFE) of a given GO term (rows) in one experimental condition (columns, WWL – WT-WT + LIF, WW – WT-Wt, WYL – WT-Y705F + LIF, WY – WT-Y705F, YYL – Y705F-Y705F + LIF, YY – Y705F-Y705F). The Fold Enrichment is the ratio of the observed frequency of a GO term to the expected frequency expected by chance. The GO term frequency was measured in the sets of proteins uniquely identified in one experimental condition that were known STAT3 targets according to the DoRothEA database. All shown GO terms had a frequency distribution across the 6 experimental conditions that was significantly deviated from an independence null hypothesis (Fisher Exact Test, p < 0.05). The heatmap is divided in three blocks corresponding to the GO domains Biological Process, Cellular Component and Molecular Function. Within each block, the order of the GO terms was determined by hierarchical clustering of their logFE profiles. A similar analysis was conducted for proteins uniquely identified in one experimental condition that were known USTAT3 targets (according to ChIP-seq data).

## Electronic supplementary material

Below is the link to the electronic supplementary material.

Supplementary Material 1

## Data Availability

Our raw data are available upon request. Proteomics data is available at https://zenodo.org/doi/10.5281/zenodo.8328034. All STAT3 constructs are deposited in Addgene plasmid repository and will be released upon publication of these results.

## Non-standard abbreviations

BiFC: Bimolecular Fluorescence Complementation
FBS: Fetal Bovine Serum
HIV: Human Immunodeficiency Virus
LIF: Leukemia Inhibitory Factor
PTM: Post-translational modification
RelA: p65 subunit of the NFkB transcription factor
RT: Reverse transcriptase
STAT3: Signal Transducer and Activator of Transcription 3
WT: Wild-type
