# Supplementary material for: Asymmetric post-translational modifications regulate the nuclear translocation of STAT3 homodimers in response to leukemia inhibitory factor
Source: Cell Oncol (Dordr). 2023 Dec 27;47(3):1065–70. doi: 10.1007/s13402-023-00911-9 (PMC11219437; doi:10.1007/s13402-023-00911-9)
Supplement: Supplementary file 1 — Supplementary Material 1 [file 13402_2023_911_MOESM1_ESM.docx]

**Supplementary Figure and Methods**

**
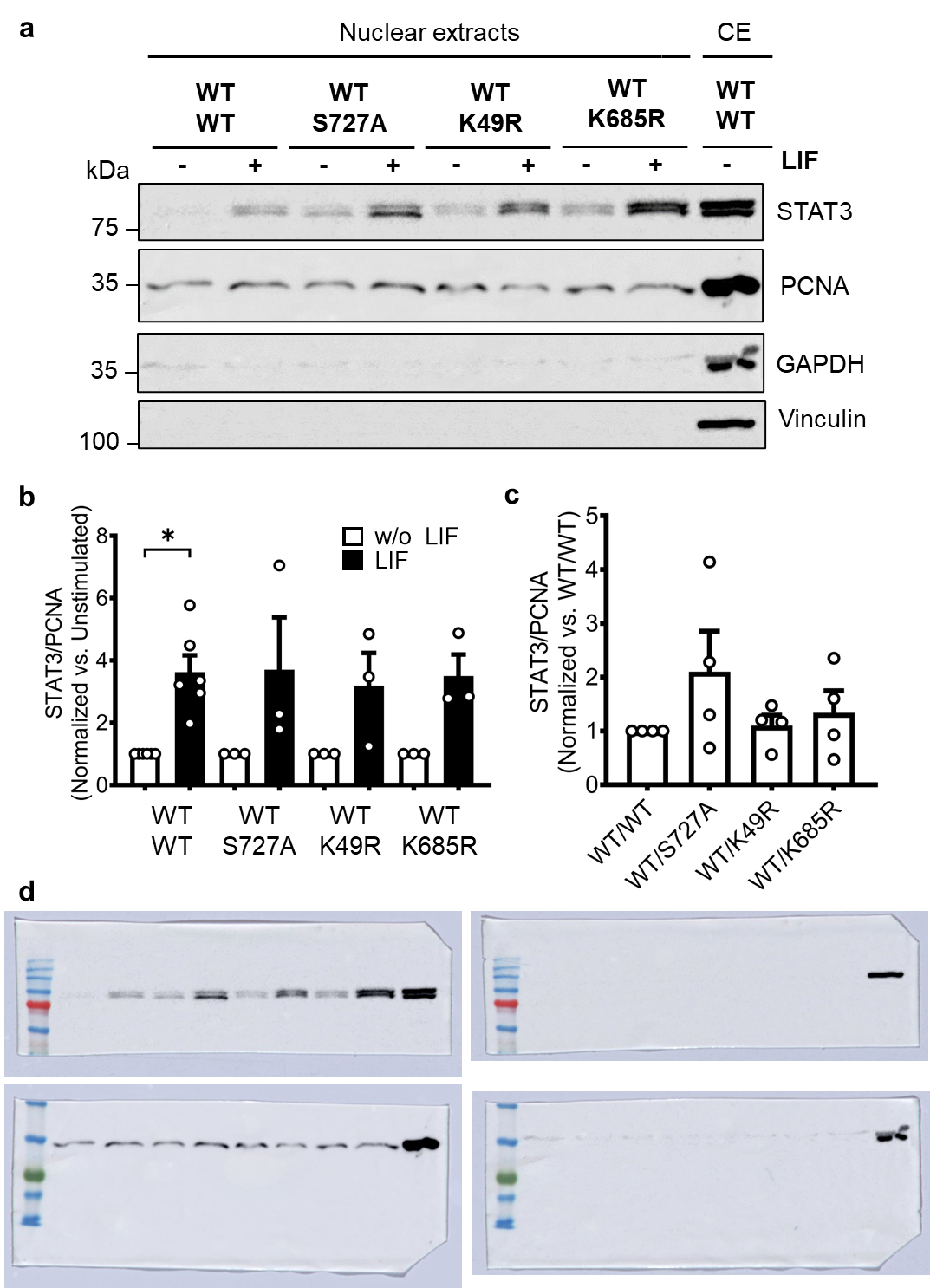
**

Supplementary Figure 1. **Preventing single PTMs in one STAT3 monomer do not block the response of STAT3 homodimers to LIF.** A, Representative images of STAT3 immunoblotting bands from cytoplasmic and nuclear protein extracts from HeLa STAT3-/- cells transfected with same DNA amounts (1 µg) of the indicated Venus-STAT3 BiFC constructs. GAPDH and Vinculin were included in the analysis to confirm the enrichment of nuclear extracts without cytosolic contamination. A lane with a cytosolic extract from cells transfected with the WT+WT combination but no stimulation with LIF was included as a positive control for cytosolic proteins. PCNA levels were used as loading control. B, Quantification of STAT3 levels, which were normalized versus PCNA levels, and then versus the ratios in unstimulated cells carrying the same combination of constructs. Results were analyzed by means of a One-way ANOVA followed by a Tukey test. *, significant, p<0.05. C, Analysis of STAT3 levels (normalized versus PCNA levels) in unstimulated cells carrying the corresponding combinations (normalized versus the WT+WT combination). Data in B and C are shown as average ± SEM of at least 3 independent experiments. D, Full membranes of the representative images, together with the molecular weight marker.

**Material & Methods**

*Cell cultures and generation of STAT3-negative HeLa cells*

HeLa cells were were a kindly gift (reference CRM-CLL-2). A STAT3 knockout (KO) strain from HeLa cells was generated using a commercial STAT3 CRISPR/Cas9 knockout plasmid kit (sc-400027, Santa Cruz Biotechnologies, Dallas, TX), following manufacturers´ instructions. Transfected cells were isolated by fluorescence-activated cell sorting (FACS) (BD FACSAria III sorter) and single cells were plated in 96-well plates for clonal expansion. Resulting clones were probed for STAT3 expression by western blot, and one of them was randomly chosen for our studies. Reference HeLa and STAT3 KO cells were maintained in Dulbecco´s Minimal Essential Medium (DMEM, Gibco, Invitrogen, Barcelona, Spain) supplemented with 10% fetal bovine serum (Biowest, Nuaillé, France) and 1% of a penicillin/streptomycin commercial antibiotic mixture (Gibco, Invitrogen, Barcelona, Spain), lines were grown to confluence in DMEM supplemented with 10% (v/v) FBS, 1% (v/v) Pen/Strep under controlled conditions of temperature and CO_2_ (37ºC, 5% CO_2_). Wild-type HeLa and STAT3-KO cell lines were periodically tested for mycoplasma contamination by means of a commercial test (Biontex Laboratories, Munich, Germany). Cell culture dishes were purchased from Techno Plastic Cultures (AG, Switzerland) unless otherwise indicated. After seeding, cells were then allowed to adhere for 16-24 h before transient transfections with the corresponding constructs. Sixteen hours later, FBS-containing medium was substituted by serum-free medium 2 hours before addition of Leukemia Inhibitory Factor (LIF, 200 ng/ml).

*Plasmids*

Venus-STAT3α BiFC constructs were described elsewhere (Letra‐Vilela et al., 2019). PTM-resistant constructs were produced by PCR-based site-directed mutagenesis using these original Venus-STAT3 constructs as templates. Table I shows the primers used for cloning and mutagenesis. All constructs were deposited in Addgene (<https://www.addgene.org/>). The original lysine (K) residues on positions 49 and 685 were replaced by arginine (R) residues, the tyrosine (Y) residue on position 705 by phenylalanine (F) and the serine (S) residue on position 727 by alanine (A). Transient transfections were carried out by means of JetPrime (Polyplus-transfection, Illkirch, France) following manufacturer´s instructions. In brief, pairs of constructs were diluted in transfection buffer prior to addition of transfection reagent. Transfection solution was added to the culture medium containing FBS (10%). After 16 hours, culture medium was removed and replaced by FBS-free medium.

Table I

| PTM mutation | | Primer sequence (5’→ 3’) |
| --- | --- | --- |
| K49R | \| FWD CATATGCGGCCAGCAGAGAATCACATGCCAC \| \| --- \| \| REV GTGGCATGTGATTCTCTGCTGGCCGCATATG \| | |
| K685R | \| FWD GAGGCATTCGGAAGGTATTGTCGGCC \| \| --- \| \| REV GGCCGACAATACCTTCCGAATGCCTC \| | |
| Y705F | \| FWD CAGGTAGCGCTGCCCCATTCCTGAAGACCAAGTTTATC \| \| --- \| \| REV GATAAACTTGGTCTTCAGGAATGGGGCAGCGCTACCTG \| | |
| S727A | \| FWD CATTGACCTGCCGATGGCACCCCGCACTTTAGATTC \| \| --- \| \| REV GAATCTAAAGTGCGGGGTGCCATCGGCAGGTCAATG \| | |

*Protein extraction and Western blots*

Western blot analysis was performed as previously described (Letra‐Vilela et al., 2019). Briefly, for total protein samples, cells were lysed using NP-40 lysis buffer (150 mM NaCl, 50 mM Tris-HCl pH 7.4/7.5, 1% NP-40 v/v) supplemented with protease inhibitors (Protease Inhibitor cocktail EDTA free, abcam, Cambridge, UK) and phosphatase inhibitors (Halt Phosphatase Inhibitor Single-use cocktail, Thermo Fisher Scientifics, Waltham, MA, USA). Samples were sonicated in UP200s sonicator (Hielscher Ultrasonics GmbH, Teltow, Germany) for 8 s, centrifuged at 10,000 x g for 10 min at 4 ºC and the soluble protein fraction was collected.

For cytoplasmic and nuclear extraction, cells were collected by trypsinization for 5 minutes at 37ºC. After centrifugation at 500 g at room temperature for 5 minutes, the supernatant was discarded and cells were resuspended in cytosolic lysis buffer (50 mM HEPES at pH 7.2, 2 mM EDTA, 10 mM NaCl, 250 mM sucrose, with 2 mM DTT, 0.1% Nonidet P-40, protease inhibitors, and phosphatase inhibitors), followed by a 5-minute incubation on ice. All centrifugations from here on were at 4ºC. Lysates were centrifuged at 3000 g for 5 minutes and the supernatant (i.e. cytosolic extract) was collected into a clean microcentrifuge tube. The pellet underwent three washes with 150 μL of cytosolic lysis buffer to remove cytosolic contaminants, and resuspended in 100 μL of nuclear lysis buffer (50 mM HEPES at pH 7.2, 2 mM EDTA, 400 mM NaCl, 20% glycerol, with 2 mM DTT, protease inhibitors, and phosphatase inhibitors). Nuclei were incubated for 40 min in ice with vortexing every 5-minutes. Subsequent centrifugation at 10000 g for 10 minutes allowed the collection of the supernatants (i.e. nuclear proteins) to a new microcentrifuge tube.

All protein extracts were quantified before their storage at -80 ºC by the Bradford method, adding 200 μL of Bradford solution (Alfa Aesar, Ward Hill, MA, USA) per 1 μL of sample for 5 min and reading absorbance at 595 nm. Serial concentrations of Albumin (0.1-2 µg of protein) were used as a standard to calculate the concentration of proteins in the samples. Forty micrograms of total protein extracts were solved by SDS-PAGE on 10% (w/v) polyacrylamide gels and transferred to a nitrocellulose membrane. Protein transfer quality was assessed by Ponceau S staining. Membranes were blocked and probed with primary antibodies in Bovine Serum Albumin (BSA) 5% (v/v) overnight at 4ºC (anti-STAT3 from Mouse at 1:1000, sc8019, Santa Cruz Biotechnology, Dallas, TX, USA; anti-GAPDH at 1:2000, from Rabbit, sc-25778, Santa Cruz Biotechnology, Dallas, TX, USA). Membranes were then washed three times with TBS-T for 10 min, followed by incubation with HRP-conjugated secondary antibody anti-Rabbit (A16096, Thermo Fisher Scientific, Waltham, MA, USA) or anti-Mouse (A16066, Thermo Fisher Scientific, Waltham, MA, USA) at dilution of 1:10000 in blocking solution (5% w/v milk in TBS-T) for 2 hours. After washing membranes three times with TBS-T for 10 min, chemiluminescence detection was performed using the Pierce ECL Plus Western Blotting Substrate and the Amersham Imager 680 blot and gel imager (Cytiva).

*Microscopy*

For microscopy, HeLa cells were seeded on glass-bottom 35 mm dishes at the density of 100.000 cells/ dish (10 mm glass surface diameter, IBIDI, Gräfelfing, Germany). Cells were transfected 16 to 24 hours after seeding with pairs of STAT3 plasmids, each containing the sequence encoding for the complementary Venus fragment. Finally, cells were starved by FBS removal from the medium 16 hours after transfection for 2 hours, before imaging. LIF treatment at 200 ng/ml is performed at t=0 of the time-lapsed acquisitions. Imaging was performed using a TCS SP8 (Leica Microsystems, Germany) widefield fluorescent microscope equipped with a Orcha-Flash4.0 Monochrome CMOS camera (Hamamatsu, Japan), a 63X NA 1.4 oil immersion objective (HC Plan Apo) and a Filter cube TRITC: YFP, ex: 517-562 nm, em: LP 590 nm. During imaging, cells were maintained at 37ºC and 5% CO_2_ conditions. Time-lapse videos were done using the Leica X Core software, and analysis was done using the FIJI free software (https:// imagej.nih.gov/ij/) and were composed of series of twenty-one images, one acquisition every minute. Kinetic analysis of time-lapse videos was carried out by measuring the fluorescence intensity in identically shaped and sized region of interest (ROI) within both nuclear and cytosol compartments of each cell. The kinetic evolution of the ratio of nuclear/cytosolic fluorescence intensity values serves as a relevant indicator of STAT3 nuclear translocation. An identically shaped and sized ROI - within a region with no apparent fluorescence detected – was used to measure the background value. Background value was then subtracted from nuclear and cytosolic values, before normalization (to t=0) and rationalization for each cell.

*Proteomics data analysis*

Protein extracts of cells expressing three STAT3 dimer variants (WT-WT, WT-Y705F and Y705F-Y705F) with and without LIF stimulation were analyzed by Mass Spectrometry (two technical replicates for each of the 6 experimental conditions). The lists of identified proteins are deposited in a public repository (<https://doi.org/10.5281/zenodo.8328034>). These lists were filtered to include only proteins identified with high confidence (technical replicate combined FDR q-value lower than 0.01). The intersections between the lists of high confidence proteins identified in the six experimental conditions were computed, and the subsets of proteins uniquely identified in each of the conditions were retrieved. These uniquely detected proteins could have been up-regulated specifically by the corresponding STAT3 dimer variant. However, we cannot ensure that the genes coding these proteins are direct targets of STAT3. Therefore, we checked which of these uniquely detected proteins were previously known to be targets of STAT3. For that we used two sources: 1) DoRothEA gene regulatory network (excluding targets of quality level E that are only supported by computational predictions) [Garcia-Alonso et al, 2019] and 2) unphosphorylated STAT3 (USTAT3) ChIP-seq data in HeLa cells (list of putative targets obtained from Cistrome Data Browser (<http://cistrome.org/db/#/>, CistromeDB entry: 45771) [Mei et al, 2016; Pope et al., 2014]. To check if the genes targeted specifically by one of the STAT3 dimer variants, with or without LIF, had similar or distinct functional profiles, we retrieved their GO annotations using the GOfuncR R package [Grote S (2023)] and applied a Fisher Exact test to detect significant associations between GO terms and subsets of experimental conditions. Associations were considered significant with p<0.05. Only GO terms associated with 4 or more proteins across the 6 experimental conditions lists were tested to ensure precision in the Fisher Exact test p value estimation. Heatmaps describing the associations of GO terms with individual experimental conditions were drawn with the ComplexHeatmap R package [Gu Z et al., 2016].

*Statistics*

Statistical analysis and graphical representation of data were performed using GraphPad Prism 9.0.0 software (GraphPad, San Diego, CA, USA). Sample data are represented as mean or violin distribution of at least three independent experiments. For statistical evaluation, results were analyzed by means of One-way ANOVA, followed by a Tukey *post-hoc* test. n=number of analyzed cells: WT pair, 160; Y705F pair, 87; WT+Y705F, 56; WT+Y705F/K49R, 59; Y705F+Y705F/K49R, 100; Y705F/K49R+Y705F/K49R, 68; WT+Y705F/K685R, 64; Y705F+Y705F/K685R, 56; Y705F/K685R+Y705F/K685R, 60; WT+Y705F/S727A, 47; Y705F+Y705F/S727A, 52; Y705F/S727A+Y705F/S727A, 78. *, significant vs each other, p<0.05; #, significant vs Y705F pair, p<0.05.

**References**

Garcia-Alonso L, Holland CH, Ibrahim MM, Turei D, Saez-Rodriguez J. 'Benchmark and integration of resources for the estimation of human transcription factor activities.' Genome Research. 2019. DOI: 10.1101/gr.240663.118.

Grote S (2023). GOfuncR: Gene ontology enrichment using FUNC. R package version 1.20.0. <https://doi.org/doi:10.18129/B9.bioc.GOfuncR>

Letra‐Vilela, R., Cardoso, B., Silva‐Almeida, C., Rocha, A. M., Murtinheira, F., Branco‐Santos, J., Rodriguez, C., Martin, V., Santa‐Marta, M., & Herrera, F. (2019a). Can asymmetric post‐translational modifications regulate the behavior of STAT3 homodimers? *FASEB BioAdvances*, fba.2019-00049. https://doi.org/10.1096/fba.2019-00049

Mei S, Qin Q, Wu Q, Sun H, Zheng R, Zang C, Zhu M, Wu J, Shi X, Taing L, Liu T, Brown M, Meyer CA, Liu XS. Cistrome Data Browser: a data portal for ChIP-Seq and chromatin accessibility data in human and mouse. Nucleic Acids Res. 2017 Jan 4;45(D1):D658-D662. doi: 10.1093/nar/gkw983.

Pope BD, Ryba T, Dileep V, Yue F, Wu W, Denas O, Vera DL, Wang Y, Hansen RS, Canfield TK, Thurman RE, Cheng Y, Gülsoy G, Dennis JH, Snyder MP, Stamatoyannopoulos JA, Taylor J, Hardison RC, Kahveci T, Ren B, Gilbert DM. Topologically associating domains are stable units of replication-timing regulation. Nature. 2014 Nov 20;515(7527):402-5. doi: 10.1038/nature13986.

Schindelin, J., Arganda-Carreras, I., Frise, E., Kaynig, V., Longair, M., Pietzsch, T., Preibisch, S., Rueden, C., Saalfeld, S., Schmid, B., Tinevez, J. Y., White, D. J., Hartenstein, V., Eliceiri, K., Tomancak, P., & Cardona, A. (2012). Fiji: An open-source platform for biological-image analysis. In Nature Methods (Vol. 9, Issue 7). https://doi.org/10.1038/nmeth.2019

Wei, W., Tweardy, D. J., Zhang, M., Zhang, X., Landua, J., Petrovic, I., Bu, W., Roarty, K., Hilsenbeck, S. G., Rosen, J. M., & Lewis, M. T. (2014). STAT3 signaling is activated preferentially in tumor-initiating cells in claudin-low models of human breast cancer. Stem Cells, 32(10). https://doi.org/10.1002/stem.1752
